# Thiazole-induced rigidification in substituted dithieno-tetrathiafulvalene: the effect of planarisation on charge transport properties

**DOI:** 10.3762/bjoc.11.129

**Published:** 2015-07-10

**Authors:** Rupert G D Taylor, Joseph Cameron, Iain A Wright, Neil Thomson, Olena Avramchenko, Alexander L Kanibolotsky, Anto R Inigo, Tell Tuttle, Peter J Skabara

**Affiliations:** 1 WestCHEM, Department of Pure and Applied Chemistry, University of Strathclyde, 295 Cathedral Street, Glasgow, G1 1XL, United Kingdom; 2 Institute of Physical-Organic Chemistry and Coal Chemistry, 02160 Kyiv, Ukraine

**Keywords:** non-covalent interactions, organic field effect transistor (OFET), organic semiconductors, tetrathiafulvalene, thiazole

## Abstract

Two novel tetrathiafulvalene (TTF) containing compounds **1** and **2** have been synthesised via a four-fold Stille coupling between a tetrabromo-dithienoTTF **5** and stannylated thiophene **6** or thiazole **4**. The optical and electrochemical properties of compounds **1** and **2** have been measured by UV–vis spectroscopy and cyclic voltammetry and the results compared with density functional theory (DFT) calculations to confirm the observed properties. Organic field effect transistor (OFET) devices fabricated from **1** and **2** demonstrated that the substitution of thiophene units for thiazoles was found to increase the observed charge transport, which is attributed to induced planarity through S–N interactions of adjacent thiazole nitrogen atoms and TTF sulfur atoms and better packing in the bulk.

## Introduction

The TTF moiety has received much attention in the field of organic electronics owing to its reliable redox behaviour [1], good charge transport properties [2] and scope for functionalisation [3]. This has led to widespread use of TTF-containing compounds in organic photovoltaic (OPV) devices [4–7] and in organic field effect transistors (OFETs), as both single crystals [8–9] and thin films [10–11] demonstrating charge carrier mobilities of up to 1.2 cm^2^ V^−1^ s^−1^ [2] for single crystal devices. Similarly, oligothiophenes have demonstrated excellent properties for use in both light-emitting [12] and light-harvesting devices [11,13]. Previously, our group reported a series of molecules containing various oligothiophenes fused to a TTF moiety through the central thiophene [11,14], which demonstrated thin film charge carrier mobilities of up to 8.61 × 10^−3^ cm^2^ V^−1^ s^−1^ [11]. Crystallographic studies of the largest of these structures revealed significant twisting of the terminal thiophene units in septithiophene chains, leading to weak conjugation in this part of the molecule [14]. We reasoned that by replacing the terminal thiophene unit with a thiazole moiety, we could induce rigorous planarity throughout the structure and thus improve conjugation, packing and therefore charge transport properties.

A typical synthesis of a dithienoTTF is performed convergently: by constructing a half-unit functionalised with a 1,3-dithiol-2-one, that can undergo a triethyl phosphite-mediated homocoupling to synthesise the central dithienoTTF in the final step. Herein, we present an alternative divergent route to dithienoTTFs, by utilising 4,6,4’,6’-tetrabromo-[2,2’]bis(thieno[3,4-*d*][1,3]dithiolylidene) [15] as a core and appending heterocyclic arms through microwave assisted Stille couplings. It is worth noting that the tetrabromo-dithienoTTF core is still prepared via a triethyl phosphite-mediated homocoupling, but in this divergent route valuable heterocyclic groups can be introduced at the end of the synthesis, instead of being carried through from the outset.

## Results and Discussion

**Figure 1:**
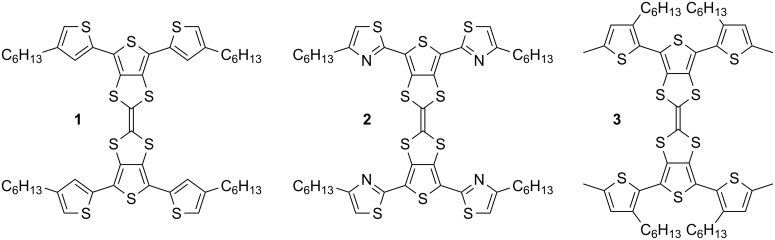
Compounds **1**–**3**.

Figure 1 shows the two novel dithienoTTFs **1** and **2** which were prepared according to the synthesis section below and studied in conjunction with a derivative **3** previously reported by our group [11].

### Synthesis

**Scheme 1:**
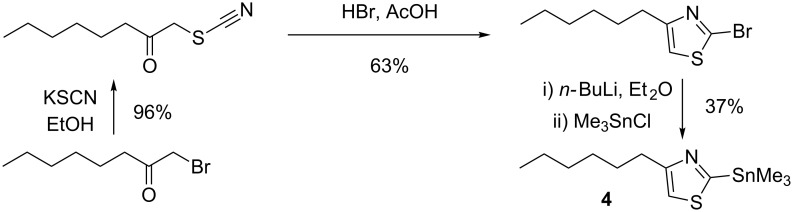
Synthesis of compound **4**.

Compounds **5** [15] and **6** [16] were prepared according to the relevant literature. Compound **4** was prepared in three steps starting with 1-bromooctan-2-one (Scheme 1). The substitution of the bromine atom for a thiocyanate group gave 1-thiocyanatooctan-2-one, which was subsequently cyclised under acidic conditions to give 2-bromo-4-hexylthiazole. Halogen–lithium exchange gave the lithiated thiazole which was quenched with trimethyltin chloride to afford **4** following purification via distillation.

**Scheme 2:**
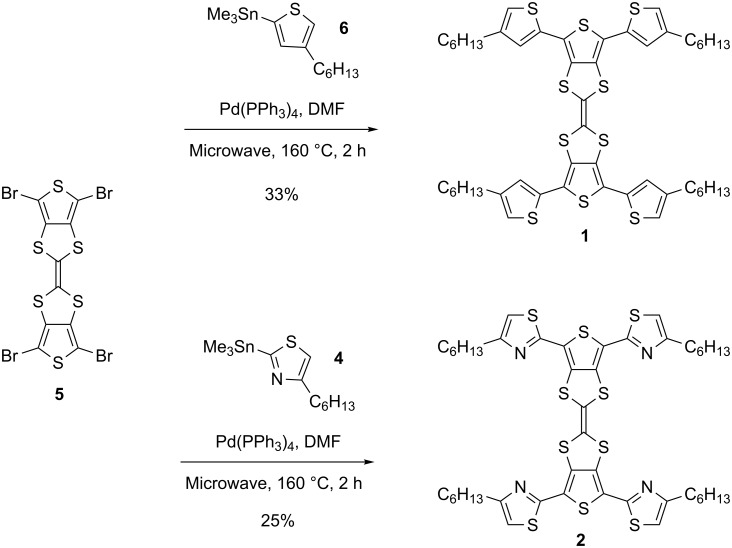
Synthesis of compounds **1** and **2**.

Initial attempts at coupling **5** with **4** or **6** by using conventional heating methods proved ineffective due to the poor solubility of **5** in common organic solvents. However, under microwave conditions successful couplings could be readily achieved in dimethylformamide (DMF) at 160 °C (Scheme 2). It is noteworthy that in both cases no partially substituted TTFs were observed as reaction byproducts (due to the incomplete reaction of all the bromine atoms), and this is attributed to the increased solubility of the intermediates, which in turn increases their statistical likelihood of reacting further.

### Optical and electrochemical properties

**Figure 2:**
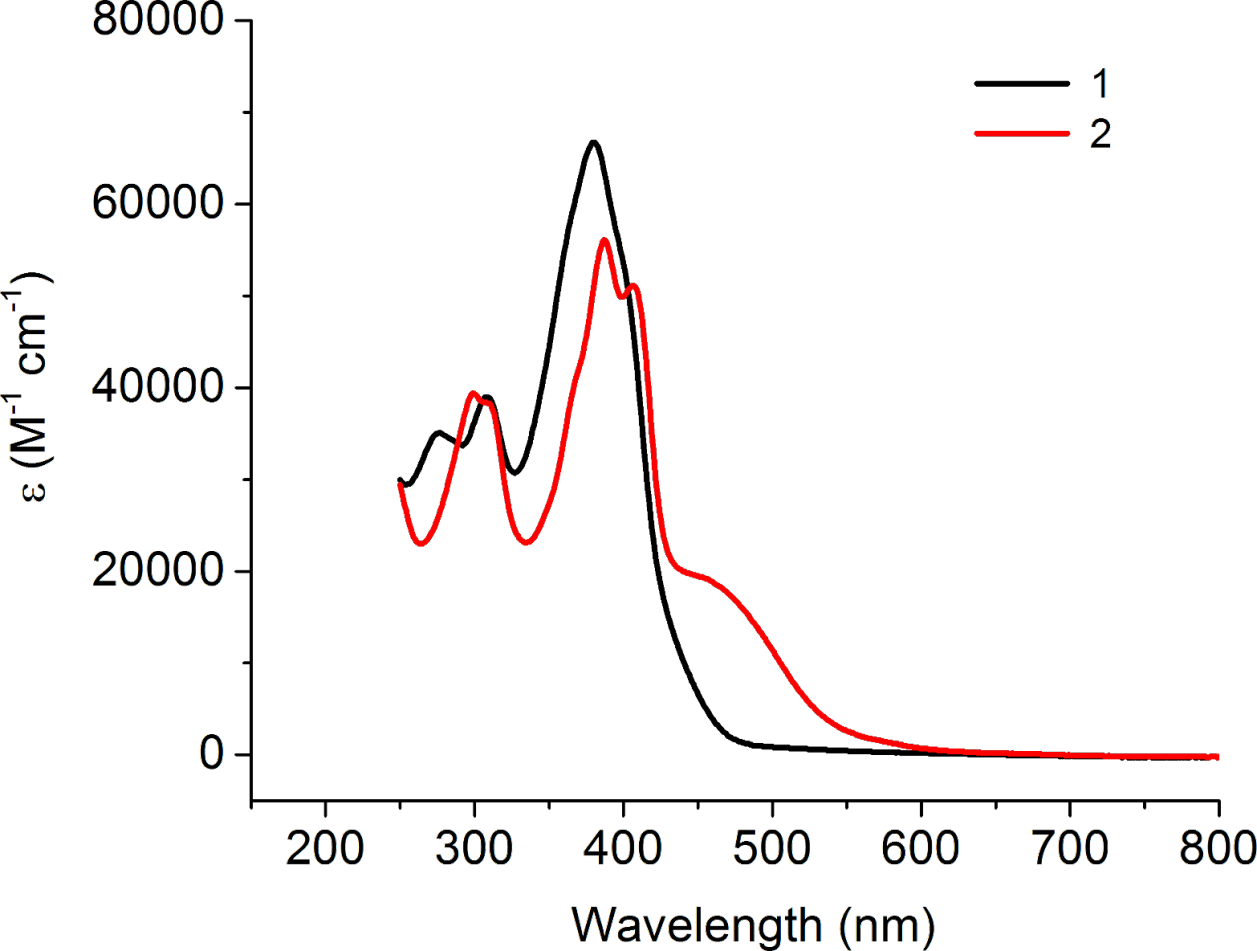
UV–vis absorption spectra of 10^−5^ M solutions of compounds **1** (black) and **2** (red) in dichloromethane.

The optical properties of TTFs **1** and **2** were analysed by solution state UV–vis spectroscopy using dichloromethane (DCM) as the solvent (Figure 2). The obtained spectrum of compound **1** shows a maximum absorption at 380 nm and two less intense peaks at 308 nm and 274 nm. The most intensive absorption peak of **2** shows a 7 nm bathochromic shift compared to **1** and this main peak shows vibronic splitting, suggesting that compound **2** is planar, induced by S–N interactions. The main peak of compound **1** also has a slight shoulder suggesting a very small amount of vibronic splitting. There is also a broad shoulder present in the spectrum for compound **2** at 454 nm, showing evidence of charge transfer (CT) from the TTF to the electron-deficient thiazole units, facilitated due to their planarity. The structural properties of each compound will be expanded upon in the theoretical calculations section.

**Figure 3:**
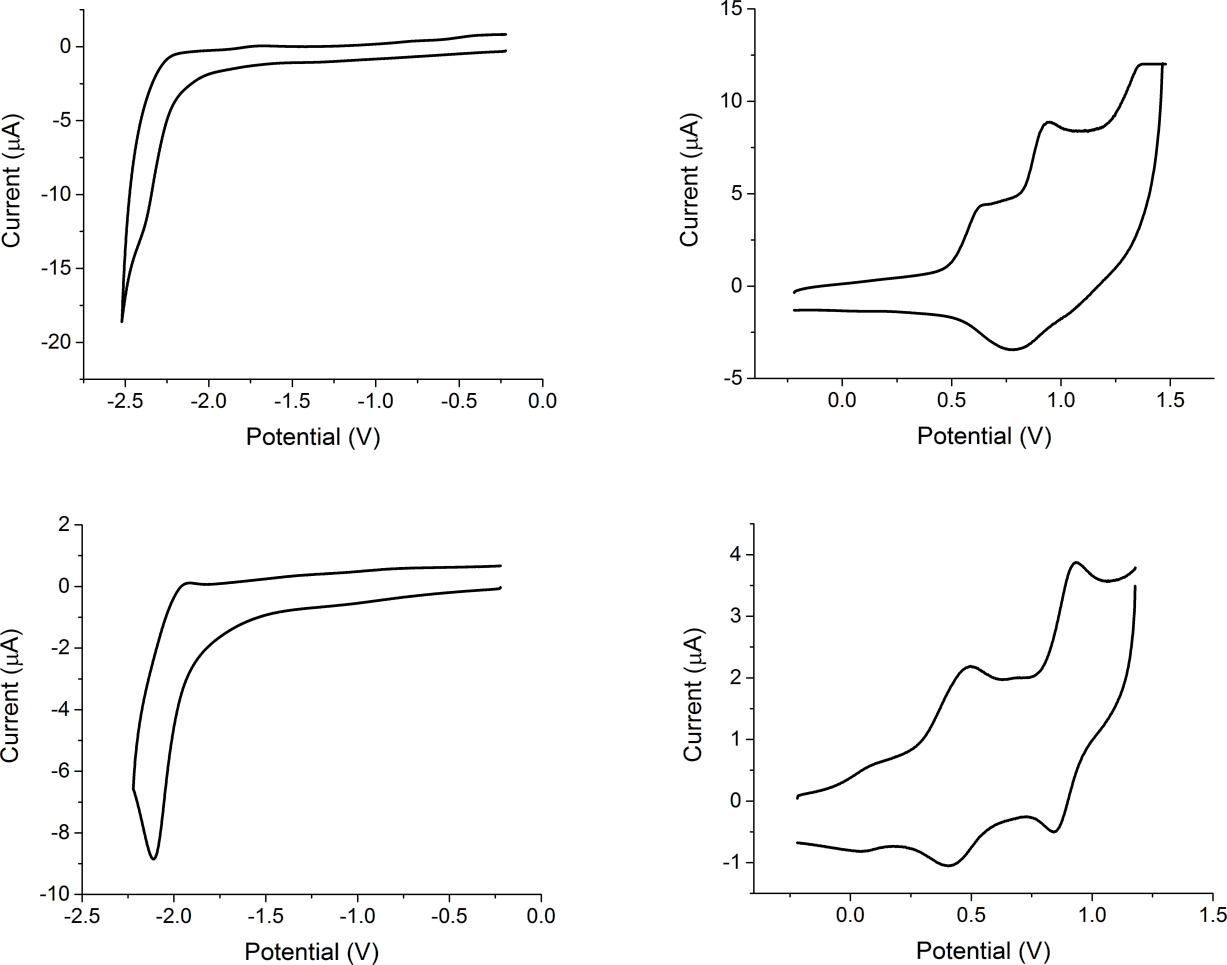
Cyclic voltammograms showing the reduction (left) and oxidation (right) of compounds 1 (top) and 2 (bottom) as 10^-4^ M solutions in dichloromethane.

Solution-state cyclic voltammetry was employed to determine the highest occupied molecular orbital (HOMO) and lowest unoccupied molecular orbital (LUMO) energy levels of TTF derivatives **1** and **2** (Figure 3). Full details of the measurements are given in Supporting Information File 1 and the results are summarised in Table 1.

**Table 1:** Summary of optical and electrochemical properties of compounds **1**–**3**.

Compound	λ_max_ (nm)	HOMO–LUMO gap (eV)^a^	HOMO (eV)^b^	LUMO (eV)^b^	*E*_ox_ (V)^c^	*E*_red_ (V)^c^

**1**	380	2.68 (2.86)	−5.30	−2.62	0.63, ir 0.94/0.78, qr 1.36/1.26, qr	−2.41, ir
**2**	387	2.22 (2.26)	−5.09	−2.87	0.49/0.41 0.93/0.84	−2.11/−1.94, ir
**3** [11]	350	2.14 (2.92)	−5.06	−2.92	0.39/0.32 0.86/0.75 1.13/1.02, qr	−2.12, ir

^a^The electrochemical HOMO–LUMO gap calculated from the difference in HOMO and LUMO energy levels. The optical HOMO–LUMO gap is calculated from the onset of absorption and is shown in parentheses. ^b^HOMO(LUMO) calculated from *E*^HOMO(LUMO)^ = (−4.80 −*E*_onset_^ox(red)^). ^c^The cathodic and anodic peaks are reported for reversible and quasi-reversible (qr) waves. The peak values on both forward and reverse scans are reported for reversible and quasi-reversible (qr) waves. The peak value on forward scan is shown for irreversible (ir) waves. The peak values are referenced to Fc/Fc^+^.

The reduction waves of compounds **1** and **2** show two irreversible peaks. DFT calculations (Supporting Information File 1, Figure SI1) indicate that the reduction of these molecules primarily takes place across the triaryl units of the substituted TTF structures, with little involvement of the electron-rich TTF moiety. The LUMO energy level of compound **2** (−2.87 eV) is 0.25 eV lower than for compound **1** (−2.62 eV), which can be explained by the incorporation of electron-deficient thiazole units leading to the stabilisation of the LUMO orbital.

The oxidation of compound **1** shows an irreversible wave followed by two quasi-reversible waves. The three anodic peaks are well resolved but there is a non-resolved peak caused by overlap of the cathodic peaks on the reverse scan. Compound **2** appears to show a first oxidation wave at 0.09 V, however, this is an artefact due to a minor degree of aggregation induced by π–π stacking between some of the molecules. This is followed by two quasi-reversible waves with well-resolved peaks. DFT calculations (Supporting Information File 1, Figure SI1) show that the HOMO is localised on the central TTF unit suggesting that this is where the first oxidation of compounds **1** and **2** will occur. The HOMO of compound **2** (−5.09 eV) was found to be 0.21 eV higher than the HOMO calculated for compound **1** (−5.30 eV). Given that TTF **2** contains electron-deficient thiazole units, it might be expected that the HOMO is lower (further from the vacuum) than that of compound **1**, however this is not the case. The increased HOMO of **2** is due to S–N interactions, resulting in donation from the nitrogen lone pair to the sulfur atoms of the TTF unit increasing the electron density on the TTF unit and destabilising the HOMO.

### Theoretical calculations

**Figure 4:**
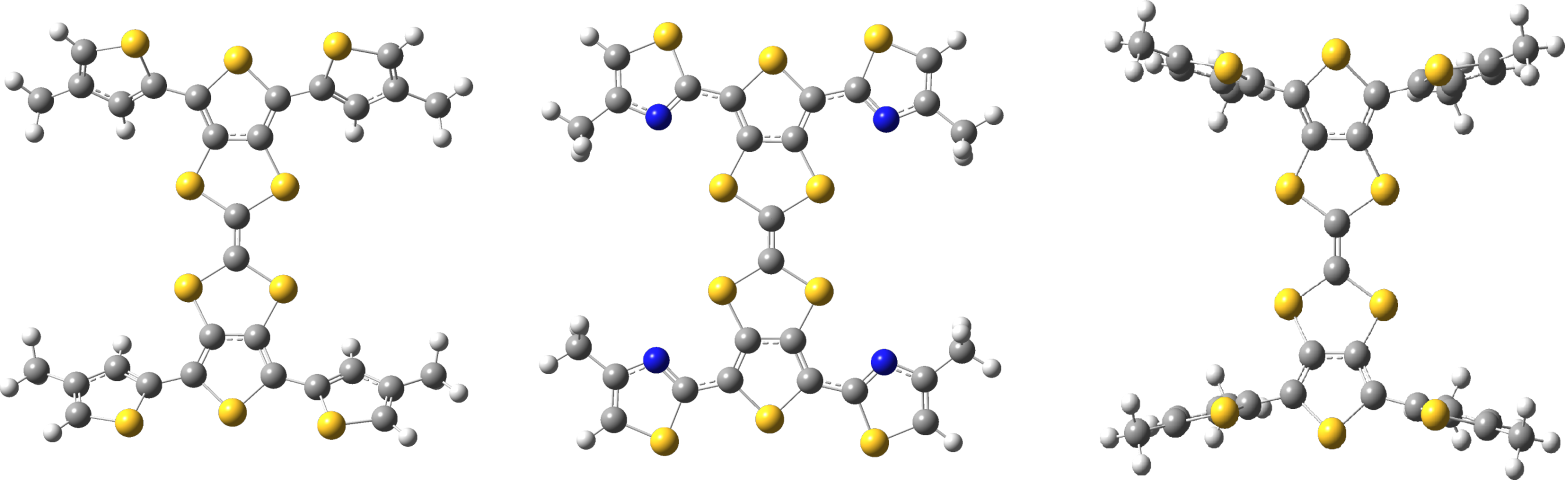
Optimised structures of **1** (left), **2** (centre) and **3** (right).

DFT calculations were carried out using the CAM-B3LYP [17] functional and TZVP [18] basis set in dichloromethane with the SMD [19] solvent model implemented in Gaussian 09 [20]. In order to reduce computational cost, hexyl chains of **1**–**3** were reduced to methyl groups (Figure 4). The dihedral angle between the peripheral thiophene and TTF units in **3** is 62°, with the presence of the alkyl chain causing inter-ring twisting due to steric hindrance. This twist is lessened when the alkyl chain is placed in the 3-position, with **1** showing a 33° twist with respect to the central TTF unit. However, replacing the thiophene units of **1** and **3** with a thiazole in **2** leads to a structure with a dihedral angle of just 5°, suggesting the influence of favourable S–N interactions contributes to a more planar structure. The S and N atoms are separated by a distance of 3.01 Å, 0.34 Å shorter than the sum of the van der Waals radii of the two atoms (3.35 Å).

In the electrochemistry section of this paper we discussed how the S–N interactions cause destabilisation of the HOMO of **2**. The HOMO energies determined from the DFT calculations (Supporting Information File 1, Figure SI1) show that the HOMO of **1** (−6.61 eV) is lower in energy that that of **2** (−6.50 eV), which is consistent with the observed experimental trend.

### Organic field-effect transistors (OFETs)

**Figure 5:**
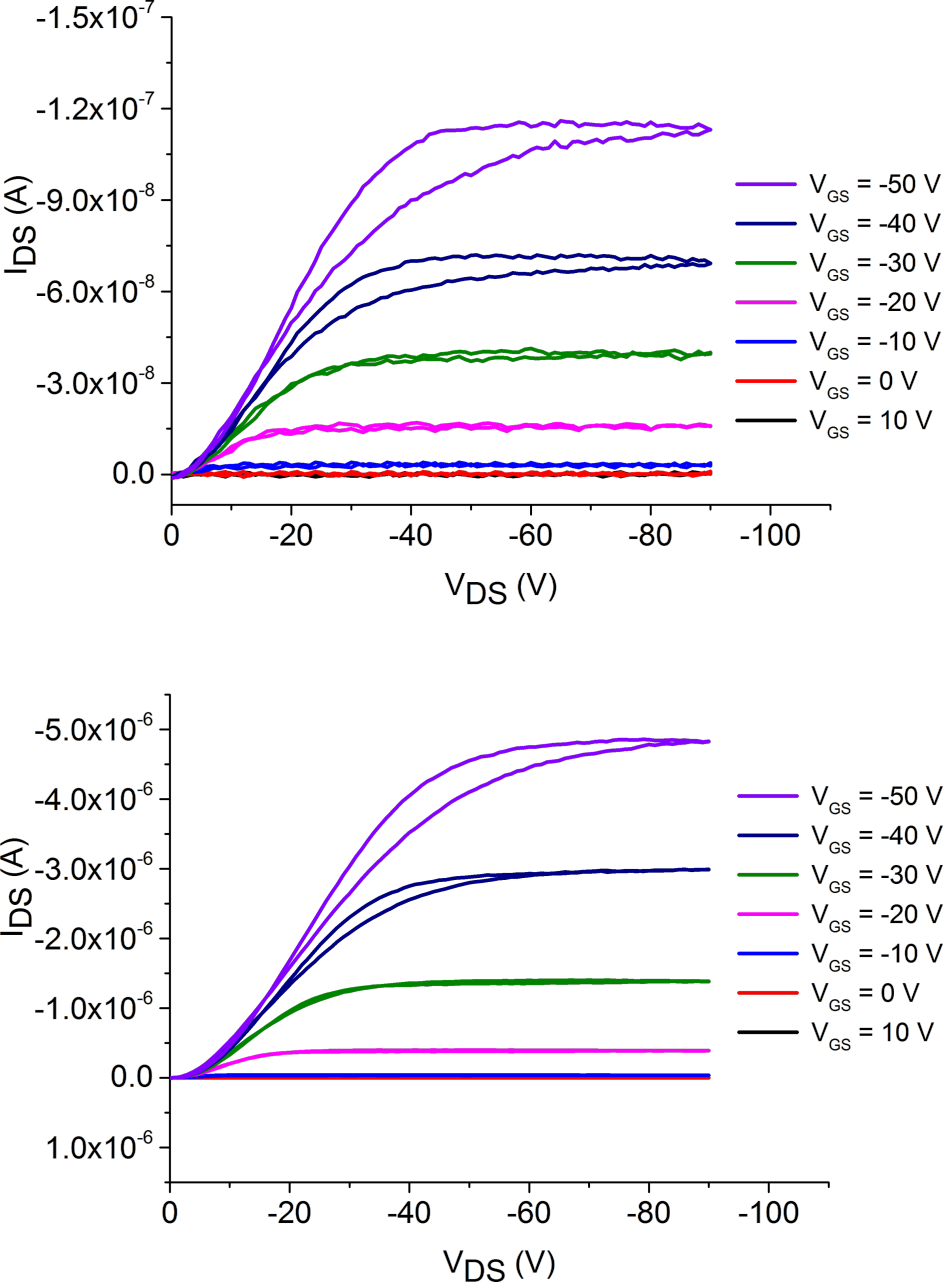
Output characteristics of OFETs fabricated using compound **2** in CHCl_3_ with OTS (top) and PFBT/OTS (bottom) as self-assembled monolayers.

Compounds **1** and **2** were used in the fabrication of bottom-gate bottom-contact OFETs to give an indication on the applicability of these small molecules to organic semiconductor devices. The use of self-assembled monolayers (SAMs) was investigated with pentafluorobenzenethiol (PFBT) and octadecyltrichlorosilane (OTS), and chloroform and *o*-dichlorobenzene were compared for solution processing of the TTF derivatives. The output and transfer characteristics for devices fabricated using compound **2** with OTS and PFBT/OTS SAMs are shown in Figure 5 and Supporting Information File 1, Figure SI2, respectively and the results are summarised in Table 2.

**Table 2:** OFET data produced from devices using CHCl_3_ as the solvent for spin coating TTF **2**.

Compound	Self-assembled monolayers	µ_h_ (cm^2^ V^−1^ s^−1)^	ON/OFF ratio	V_T_ (V)

**2**	OTS	1.35 × 10^−5^	10^1^	−30
	PFBT/OTS	3.47 × 10^−4^	10^3^	−34

The OFETs fabricated using only OTS as the SAM from a chloroform solution show an average saturation hole mobility of 1.35 × 10^−5^ cm^2^ V^−1^ s^−1^ and an ON/OFF ratio of 10^1^. However, the use of both PFBT and OTS SAMs and a chloroform solution of compound **2** results in a mobility of 3.47 × 10^−4^ cm^2^ V^−1^ s^−1^, an increase of an order of magnitude. An increase is also observed in the ON/OFF ratio by two orders of magnitude to 10^3^. There was no observed field-effect for devices fabricated using compound **2** in an *o-*dichlorobenzene solution or any OFETs fabricated using compound **1**.

**Figure 6:**
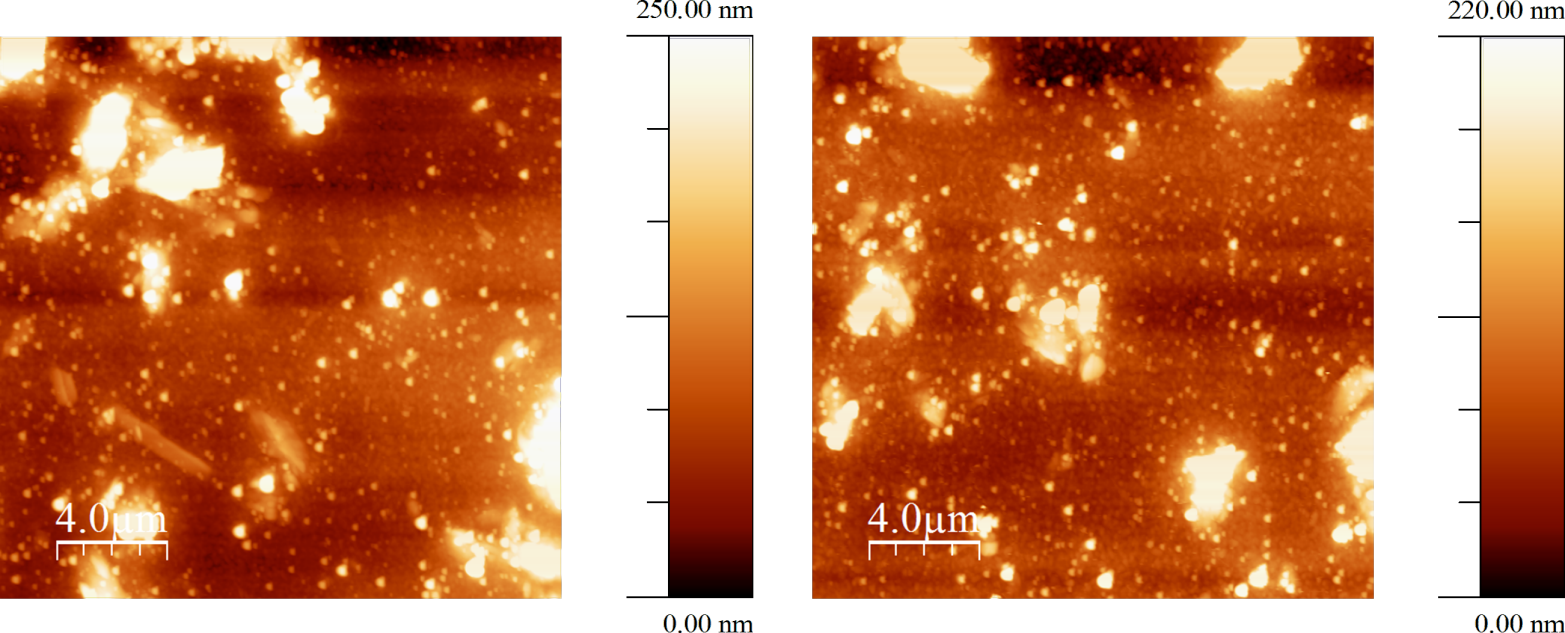
AFM images of OFET devices fabricated using compound **2** in CHCl_3_ with OTS (left) and PFBT/OTS (right) as the SAMs.

Atomic force microscopy (AFM) was used in order to analyse the surfaces of the devices fabricated. Shown below in Figure 6 are images from devices containing compound **2** with OTS and PFBT/OTS SAMs. The images look similar with a combination of small and large clusters covering the surface. This suggests that the improvement in OFET performance with the addition of PFBT is due to improved charge injection, rather than an improvement in the morphology of the film. The OFETs were tested for n-type mobility but there was no field-effect observed.

Interestingly, despite OFETs fabricated from compound **1** showing no field-effect, films formed using this compound show a good degree of order. Figures SI3 and SI4 in Supporting Information File 1 show the surfaces of OFETs made using solutions of compound **1** in *o-*dichlorobenzene and chloroform, respectively. The film formed from spin-coating the *o-*dichlorobenzene solution shows the formation of long needle-like structures, whilst the film that results from spin-coating from the chloroform solution is less thick and shows needle-like structures which are shorter and wider. An explanation for the fact that there was no observed field-effect could be due to limited charge transport between the needles in these films.

## Conclusion

By successfully coupling the tetrabrominated dithienoTTF (**5**) with stannylated intermediates **4** and **6** (via a four-fold Stille coupling), we have presented a novel route to substituted dithienoTTFs that does not necessitate the need for the synthesis of a half-unit and a final triethyl phosphite mediated homo-coupling. The resultant compounds, **1** and **2**, were fully characterised and their optical and electrochemical properties elucidated via UV–vis spectroscopy and cyclic voltammetry, and explained in conjunction with DFT level calculations. OFETs fabricated using **1** were found to have no observable field effect, but those fabricated from compound **2** were found to sustain charge carrier mobilities up to 3.47 × 10^−4^ cm^2^ V^−1^ s^−1^. The increased performance of thiazole TTF **2** is attributed to the planarising effect brought about by S–N interactions between the thiazole nitrogen atom and TTF sulfur atoms.

## Supporting Information

File 1 Full experimental and characterisation for compounds **1**, **2** and **4**–**6**, as well as OFET fabrication methods and AFM images of OFETs containing **2**.
