# Supplementary material for: Thiazole-induced rigidification in substituted dithieno-tetrathiafulvalene: the effect of planarisation on charge transport properties
Source: Beilstein J Org Chem. 2015 Jul 10;11:1148–54. doi: 10.3762/bjoc.11.129 (PMC4505100; doi:10.3762/bjoc.11.129)
Supplement: File 1 — Full experimental and characterisation for compounds 1, 2 and 4–6, as well as OFET fabrication methods and AFM images of OFETs containing 2. [file Beilstein_J_Org_Chem-11-1148-s001.pdf]

Supporting Information

for

Thiazole-induced rigidification in substituted dithieno-tetrathiafulvalene: the effect of planarisation on charge transport properties

Rupert G. D. Taylor[1], Joseph Cameron[1], Iain A. Wright[1], Neil Thomson[1], Olena Avramchenko[1], Alexander L. Kanibolotsky[1,2], Anto R. Inigo[1], Tell Tuttle[1], Peter J. Skabara[1]*

Address: [1]WestCHEM, Department of Pure and Applied Chemistry, University of Strathclyde, 295 Cathedral Street, Glasgow, G1 1XL, United Kingdom and [2]Institute of Physical-Organic Chemistry and Coal Chemistry, 02160 Kyiv, Ukraine

Email: Peter Skabara - peter.skabara@strath.ac.uk

*Corresponding author

Full experimental and characterisation for compounds 1, 2 and 4–6, as well as OFET fabrication methods and AFM images of OFETs containing 2.

1. General remarks

$Pd(PPh_3)_4$ was prepared prior to use and stored in a freezer under nitrogen. Unless otherwise stated, all other reagents were sourced commercially and used without further purification. ^1H and ^{13}C NMR spectra were recorded on a Bruker Avance DPX400 apparatus at 400.1 and 100.6 MHz respectively or on a Bruker Avance III at 600.1 and 150.9 MHz respectively. Chemical shifts are given in ppm, all *J* values are quoted in Hz. Low resolution mass spectrometry (LRMS) was performed on a Shimadzu Axima-CFR spectrometer (MALDI). High resolution mass spectrometry (HRMS) was gratefully

performed by the EPSRC national facility at Swansea. Dry solvents were obtained from a solvent purification system (SPS 400 from Innovative Technologies). Thermogravimetric analysis (TGA) was performed using a Perkin-Elmer Thermogravimetric Analyzer TGA7 under a constant flow of argon. Melting points were recorded using a TA instruments DSC QC1000 Differential Scanning Calorimeter and are uncorrected.

Cyclic voltammetry (CV) measurements were performed on a CH Instruments 660A electrochemical workstation with iR compensation using anhydrous dichloromethane as the solvent and tetrabutylammonium hexafluorophosphate as the electrolyte at 0.1 M. The electrodes used were glassy carbon, platinum wire and silver wire as the working, counter and reference electrodes respectively. All solutions were degassed using Ar and contained the analyte compound at concentrations of ca. 10^{-4} M. All measurements were referenced against the $E_{1/2}$ of the Fc/Fc^{+} redox couple and HOMO/LUMO levels were calculated assuming a ferrocene HOMO level of −4.8 eV. Absorption spectra were recorded using a Shimadzu UV 7200 instrument.

Organic field-effect transistors were fabricated on SiO_2 substrates with prefabricated interdigitated Au source-drain channels with lengths of 2.5, 5, 10 and 20 µm and width of 1 cm. N-doped Si and SiO_2 were the gate electrode and gate dielectric, respectively. The substrates were cleaned using water, acetone and ethanol before being treated in UV-ozone for 30 seconds. The pentafluorobenzenethiol (PFBT) self-assembled monolayer (SAM) was prepared by dropcasting a solution of PFBT (10 mM in ethanol) onto the substrate. After 1 min, the residual PFBT was then washed with ethanol and the substrate was dried over a stream of compressed air. Similarly, octadecyltrichlorosilane (OTS) SAM was prepared by dropcasting an OTS solution (13 mM in toluene) onto the substrate which was washed with toluene and dried after 1 min. Devices utilising both SAMs were prepared by sequential dropcasting of the SAM solutions as above (PFBT then OTS). Compounds **4** and **5** were deposited *via* spin-coating 10 mg ml^{-1} chloroform and *o*-dichlorobenzene solutions. Films of compounds **4** and **5** were annealed at 100 °C and 120 °C respectively. Current–voltage characteristics were recorded using a Keithley 4200 semiconductor parameter analyser at room temperature in a nitrogen atmosphere. The field-effect mobilities were determined from the saturation regime and calculated using the following equation:

$$\mu_{sat} = \frac{2L}{WC_i} \times \left(\frac{\partial\sqrt{I_{DS}}}{\partial V_{GS}}\right)^2$$

The surface morphology was characterised using a Dimension 3100 atomic force microscope (AFM) in tapping mode.

2. Experimental procedures

(4-Hexylthiophen-2-yl)trimethylstannane (6)

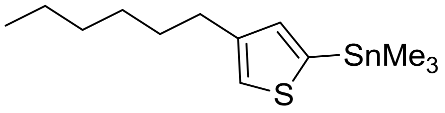

n-Butyllithium (2.4 M in hexane, 7.43 ml, 17.83 mmol) was added to a solution of 3-hexylthiophene (3.21 ml, 17.83 mmol) and tetramethylethylenediamine (2.96 ml, 19.6 mmol) in anhydrous diethyl ether (70 ml) under Ar at room temperature and then heated to reflux for 1 h. The reaction mixture was then cooled to 0 °C before trimethyltin chloride solution (1.0 M in THF, 19.6 ml, 19.6 mmol) was added, after which the reaction mixture was stirred at room temperature for 2 h. Triethylamine (10 ml) was added to quench the reaction, which was then washed with water (4 × 50 ml). The organic layer was dried over $MgSO_4$ and the solvents removed under reduced pressure to give (4-hexylthiophen-2-yl)trimethylstannane (**6**) (5.40 g, 92%) as a colourless oil, used without further purification. ^{1}H NMR (400 MHz, $CDCl_3$) δ 7.20 (d, J = 0.8 Hz, 1H, ArH), 7.01 (s, 1H, ArH), 2.64 (t, J = 8.0 Hz, 2H, CH_2), 1.63 (m, 2H, CH_2), 1.32 (m, 6H, CH_2), 0.89 (t, J = 6.8 Hz, 3H, CH_3), 0.35 (s, 9H, CH_3), consistent with the literature [1].

1-Thiocyanatooctan-2-one

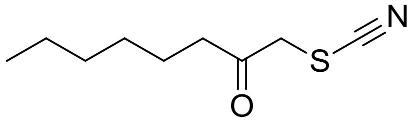

Potassium thiocyanate (2.63 g, 27.1 mmol) was added to a solution of 1-bromooctan-2-one [2,3] (4.68 g, 22.6 mmol) in ethanol (45 ml) and stirred at room temperature overnight. The solvent was removed under reduced pressure and the residue taken up into water (100 ml). The product was extracted into

ethyl acetate (3 × 50 ml), combined, washed with brine (100 ml), dried over Na_2SO_4 and concentrated under reduced pressure to give 1-thiocyanatooctan-2-one (4.00 g, 96%) as a yellowish liquid, used without further purification. ^{1}H NMR (400 MHz, $CDCl_3$) δ 4.02 (s, 2H, CH_2), 2.57 (t, J = 7.4 Hz, 2H, CH_2), 1.72 – 1.58 (m, 2H, CH_2), 1.38 – 1.22 (m, 6H, CH_2), 0.96 – 0.84 (m, 3H, CH_3).

2-Bromo-4-hexylthiazole

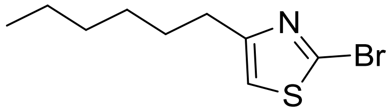

1-Thiocyanatooctan-2-one (4.00 g, 16.1 mmol) was added to a 33 % solution of HBr in acetic acid (45 ml) and stirred overnight at room temperature. The reaction mixture was then poured onto ice and neutralised carefully with NaOH keeping the temperature below 10 °C. The mixture was extracted with CH_2Cl_2 (3 × 50 ml) and the combined extracts washed with brine (100 ml) and dried over Na_2SO_4. The crude product was purified by silica gel column (hexane:CH_2Cl_2, 9:1) to give 2-bromo-4-hexylthiazole (3.40 g, 63%) as a yellowish oil. ^{1}H NMR (400 MHz, $CDCl_3$) δ 6.81 (s, 1H, ArH), 2.72 (t, J = 7.7 Hz, 2H, CH_2), 1.75 – 1.60 (m, 2H, CH_2), 1.40 – 1.22 (m, 6H, CH_2), 0.87 (t, J = 6.8 Hz, 3H, CH_3). ^{13}C NMR (101 MHz, $CDCl_3$) δ 158.5, 134.9, 116.6, 31.8, 31.7, 29.1, 29.0, 22.7, 14.2. LRMS (CI, *m/z*): 247.9 $[M+H]^+$, 275.9 $[M+C_2H_5]^+$, 289.9 $[M+C_3H_7]^+$.

4-Hexyl-2-(trimethylstannyl)thiazole (4)

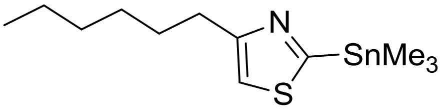

n-Butyllithium (2.35 M in hexane, 0.45 ml, 1.058 mmol) was added dropwise to a solution of 2-bromo-4-hexylthiazole (0.262 g, 1.056 mmol) in anhydrous diethyl ether (6 ml) at −80 °C under an atmosphere of nitrogen. The reaction was stirred for 1 h at −80 °C, then trimethyltin chloride solution (1.0 M in THF, 1.59 ml, 1.59 mmol) was added dropwise and the reaction warmed to room temperature and left to stir overnight. The reaction was diluted with ether (10 ml), washed with water (2 × 20 ml) and brine (20 ml), dried over $MgSO_4$ and concentrated under reduced pressure to give a

pale oil which was purified via kugelrohr distillation (removal of lower boiling impurities up to 100 °C at 0.31 mbar) to give 4-hexyl-2-(trimethylstannyl)thiazole (**4**) as a pale yellow oil (0.13 g, 37%). ^{1}H NMR (400 MHz, $CDCl_3$) δ 7.05 (m, 1H, Ar*H*), 2.90 (m, 2H, CH_2), 1.73 (m, 2H, CH_2), 1.40 – 1.29 (m, 6H, CH_2), 0.88 (m, 3H, CH_3), 0.45 (s, 9H, CH_3).

Thiophene TTF (1)

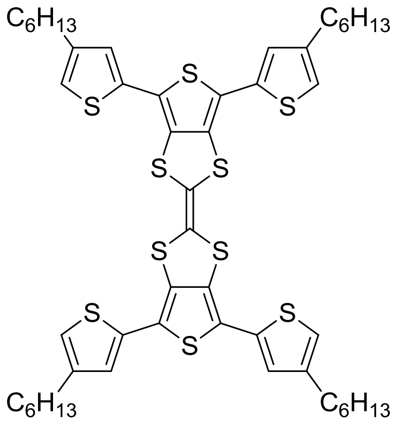

4,4’,6,6’-Tetrabromo-2,2’-bis(thieno[3,4-*d*][1,3]dithiolylidene) (**5**) (0.050 g, 0.089 mmol) and 2-trimethylstannyl-4-hexylthiophene (0.120 g, 0.363 mmol) (**6**) were added to a microwave vial which was evacuated and purged with nitrogen before the addition of anhydrous DMF (2.5 ml). The mixture was thoroughly degassed with nitrogen for 45 min before $Pd(PPh_3)_4$ (0.0183 g, 0.016 mmol) was added. The vial was sealed and heated to 160 °C for 2 h under microwave irradiation. After cooling to room temperature, the solution was poured into water (10 ml), extracted with $CHCl_3$ (3 × 10 ml), combined, washed with water (2 × 30 ml) and brine (30 ml), dried over $MgSO_4$ and concentrated under reduced pressure. The crude material was then treated with methanol and cold hexane to give thiophene-TTF (**1**) (yield 0.026 g, 33%) as a light brown solid. ^{1}H NMR (400 MHz, $CDCl_3$) δ 6.98 (s, 4H, Ar*H*), 6.92 (s, 4H, Ar*H*), 2.62 (t, *J* = 7.6 Hz, 8H, CH_2), 1.65 (m, 8H, CH_2), 1.38 (m, 24H, CH_2), 0.91 (t, *J* = 6.4 Hz, 12H, CH_3); ^{13}C NMR (101 MHz, $CDCl_3$) δ 144.7, 134.8, 131.4, 126.1, 121.9, 120.4, 119.4, 31.8, 30.6, 30.5, 29.2, 22.8, 14.3. LRMS (MALDI, *m/z*) calc. for $C_{50}H_{60}S_{10}$: 980.19 (M^+), found 980.02. HRMS (APCI, *m/z*) calc. for $C_{50}H_{61}S_{10}$ ($[M + H]^+$) 981.1975, found m/z 981.1983 ($[M + H]^+$); TGA (Argon): Initial weight loss due to thermal degradation commences at 332 °C.

Thiazole TTF (2)

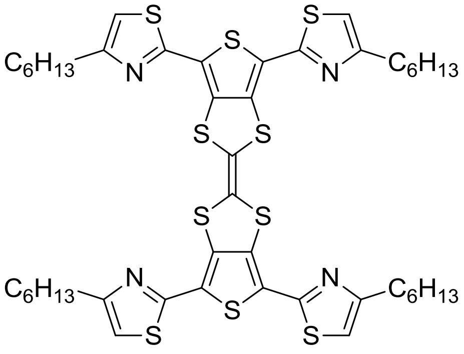

4,4',6,6'-Tetrabromo-2,2'-bis(thieno[3,4-*d*][1,3]dithiolylidene) (**5**) (0.041 g, 0.065 mmol) and 4-hexyl-2-(trimethylstannyl)thiazole (**4**) (0.099 g, 0.298 mmol) were added to a microwave vial which was evacuated and purged with nitrogen before the addition of anhydrous DMF (2 ml). The suspension was thoroughly degassed with nitrogen for 45 min before $Pd(PPh_3)_4$ (0.015 g, 0.013 mmol) was added. The vial was sealed and heated to 160 °C for 2 h under microwave irradiation. After cooling to room temperature, the solution was poured into water (10 ml), extracted with $CHCl_3$ (3 × 10 ml), combined, washed with water (2 × 30 ml) and brine (30 ml), dried over $MgSO_4$ and concentrated under reduced pressure. The crude material was then treated with methanol and cold hexane to give thiazole-TTF (**2**) (0.016 g, 25%) as a deep red powder. ^{1}H NMR (400 MHz, $CDCl_3$) δ 6.93 (s, 4H, Ar*H*), 2.85 (t, *J* = 7.6 Hz, 8H, CH_2), 1.48 – 1.31 (m, 32H, CH_2), 0.91 (m, 12H, CH_3); ^{13}C NMR (150 MHz, $CDCl_3$) δ 159.4, 158.0, 135.6, 122.9, 121.0, 113.5, 31.9, 31.6, 29.1, 29.1, 22.8, 14.3; LRMS (MALDI, *m/z*), calc. for $C_{46}H_{56}N_4S_{10}$: 984.17 (M+), found 983.96, HRMS (APCI, *m/z*) calc. for $C_{46}H_{57}N_4S_{10}$ ($[M + H]^+$) 985.1785 found 985.1786 ($[M + H]^+$). TGA (Argon): Initial weight loss due to thermal degradation commences at 320 °C.

3. Frontier Orbitals

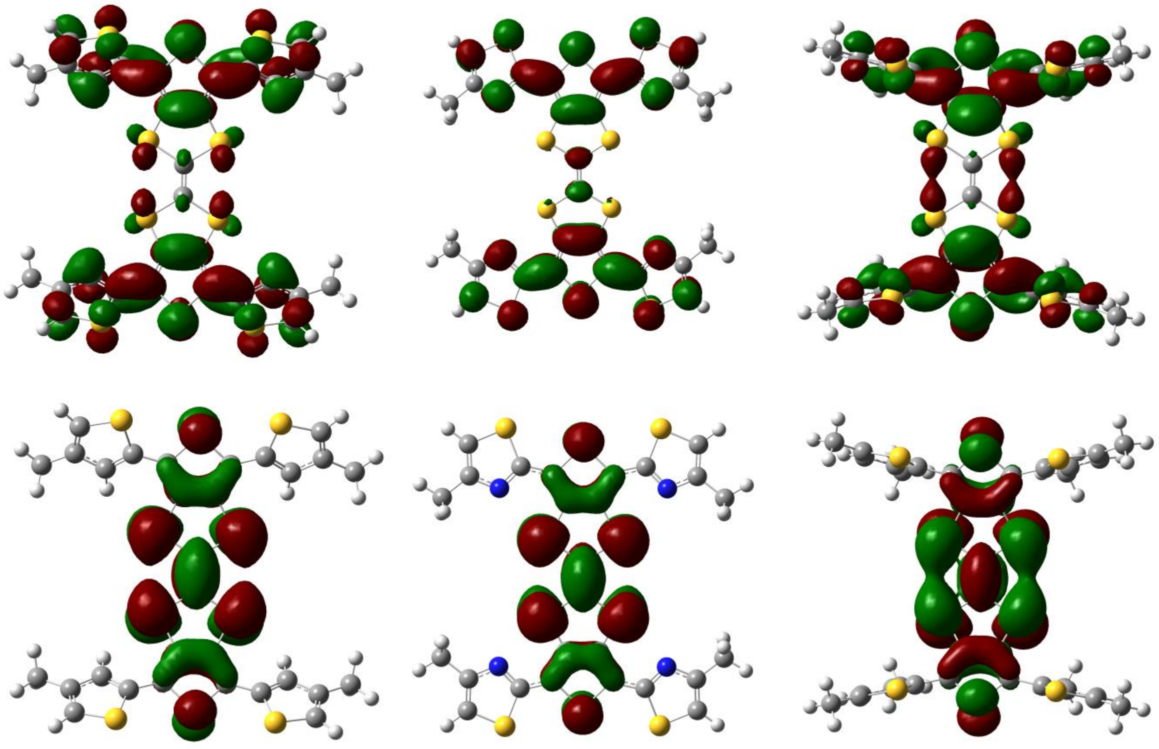

Figure SI1. HOMOs (bottom) and LUMOs (top) of compounds **1** (left), **2** (centre) and **3** (right).

4. OFET data

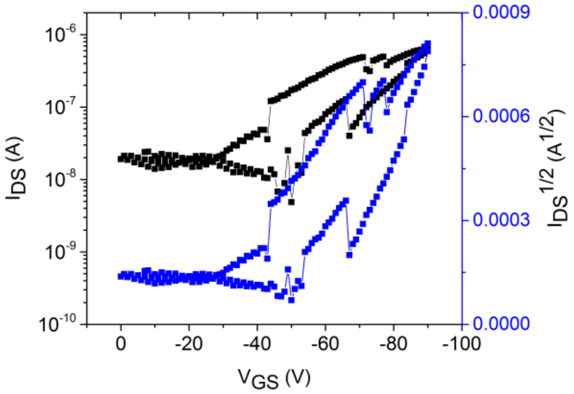

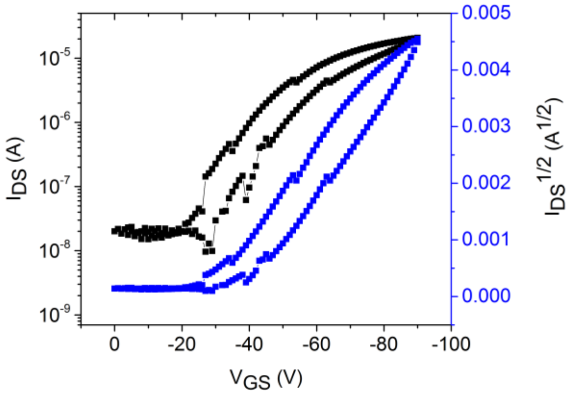

Figure SI2. Output characteristics of OFETs fabricated using compound **2** in $CHCl_3$ with OTS (top) and PFBT/OTS (bottom) as self-assembled monolayers.

5. AFM images

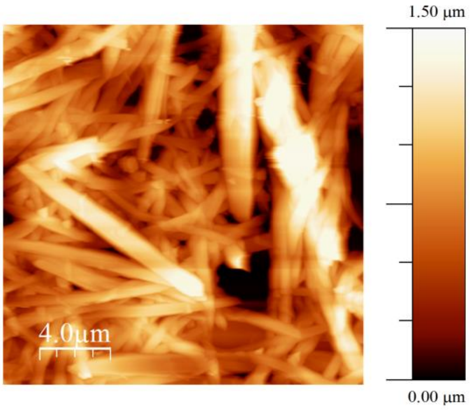

Figure SI3. AFM image of **an** OFET **device** fabricated with OTS/PFBT SAMs and compound **1** in *o*-dichlorobenzene.

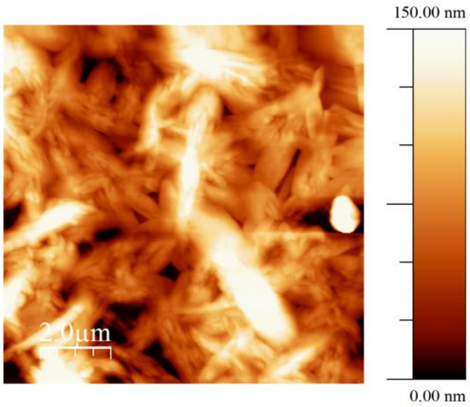

Figure SI4. AFM image of **an** OFET **device** fabricated with OTS/PFBT SAMs and compound **1** in chloroform.

6. References

1. Barbarella, G.; Bongini, A.; Zambianchi, M. *Macromolecules* **1994,** *27*, 3039. doi:10.1021/ma00089a022

2. Lee, J.; Jung, B.-J.; Lee, S. K.; Lee, J.-I.; Cho, H.-J.; Shim, H.-K. *J. Polym. Sci., Part A: Polym. Chem.* **2005,** *43*, 1845. doi:10.1002/pola.20659

3. Guo, X.; Fan, H.; Zhang, M.; Huang, Y.; Tan, S.; Li, Y. *J. Appl. Polym. Sci.* **2012,** *124*, 847. doi:10.1002/app.35106
